# Improvement in the Cognitive Aspects of Cultural Competence after Short-Term Overseas Study Programs

**DOI:** 10.3390/ijerph18137102

**Published:** 2021-07-02

**Authors:** Chen Wang, Xiang-Yu Hou, Nigar G. Khawaja, Michael P. Dunne, Jane Shakespeare-Finch

**Affiliations:** 1 Center for Brain, Mind and Education, Shaoxing University, Shaoxing 312000, China; 2 School of Teacher Education, Shaoxing University, Shaoxing 312000, China; 3 Faculty of Health, School of Psychology and Counseling, Queensland University of Technology, Brisbane 4059, Australia; n.khawaja@qut.edu.au (N.G.K.); j.shakespeare-finch@qut.edu.au (J.S.-F.); 4 School of Health and Wellbeing, University of Southern Queensland, Brisbane 4059, Australia; janet.hou@usq.edu.au; 5 Institute for Community Health Research, Hue University, Hue 47000, Vietnam; m.dunne@qut.edu.au; 6 Australian Centre for Health Law Research, Faculty of Business and Law, Queensland University of Technology, Brisbane 4059, Australia

**Keywords:** cultural competence, overseas study experiences, open-mindedness, healthcare students

## Abstract

Universities are providing short-term overseas study programs for healthcare students to increase their cultural competence (i.e., capacity to work effectively in cross-cultural situations). However, there is limited empirical research evaluating the effects of these programs using well-controlled research designs. In the present research study, undergraduate healthcare students in an Australian university were selected as participants. Group 1 (*n* = 32) participated in a short-term overseas study program in Asia (i.e., China, Vietnam, Singapore, and Taiwan), whereas Group 2 (*n* = 46) stayed in Australia to continue their university education as usual. All participants completed a self-developed demographic questionnaire, Cultural Intelligence Scale, and Multicultural Personality Questionnaire. Cultural competence was surveyed pre- and post-short-term overseas programs. After controlling for prior overseas experiences and the open-mindedness trait, an ANCOVA indicated that Group 1 had a significantly higher scores than Group 2 in cultural knowledge (*p* < 0.05), but not in cultural awareness, attitude, or skills. It is suggested that short-term overseas study programs may increase healthcare students’ cultural knowledge, a component of competence, and that more needs to be accomplished to improve other areas of cultural competence.

## 1. Introduction

The Australia National Health and Medical Research Council (NHMRC) defined cultural competence as “a set of congruent behaviours, attitudes, and policies that come together in a system, agency, or amongst professionals and enables that system, agency, or those professionals to work effectively in cross-cultural situations” [1]. In addition to this definition in the health area, general theoretical research on cultural competence has developed several models of this concept. According to the theory of cultural intelligence (CQ model), cultural competence includes metacognitive (cultural awareness), cognitive (cultural knowledge), motivational (cultural attitude), and behavioural (cultural skills) aspects [2,3]. The present study used this four-dimensional model of cultural competence.

Health professionals’ understanding of culture and their intercultural communication skills can affect the quality of healthcare delivery [4]. Specifically, the cultural background of a healthcare professional can influence how they interpret, assign meaning to, and construct value judgments about their clients [5]. There is compelling evidence that a lack of cultural competence among professionals can result in poor healthcare outcomes [6,7]. The NHMRC acknowledged the significance of cultural competence in healthcare and suggested education providers take action to encourage healthcare professionals and students to develop their cultural competence [1]. Additionally, the role of culture has been emphasised in the education of healthcare students [8].

Considering healthcare students’ career preparation, Australian universities are providing various cultural learning programs to improve their students’ potential of being more culturally competent [9]. These programs are designed according to the factors associated with cultural competence, and increasing cultural exposure is the major means that cultural learning programs work on [10,11]. According to a review study on cultural education, cultural learning programs mainly fall into three categories: in-class cultural learning, off-campus cultural-related activities, and overseas study [11]. In-class cultural learning programs often focus on teaching students cultural information related to health and wellbeing. Off-campus culture-related activities are arranged to increase cultural competence through exposure to diversity, and “intercultural networking”, that is, cross-cultural communication in their own country. Overseas study programs refer to studying abroad where students may learn via scripted programs, professional skills training in workplaces, and general experiences of daily life in the host countries.

According to the Institute of International Education in the USA, overseas study programs can be short term (no more than eight weeks) or long term, and include various types, such as international service learning, international elective courses, exchange study, international study tours, full-year programs, semester programs and summer programs [12]. Overseas study programs exist across different disciplines, but those in the area of health usually have a focus on cultural competence [11,13,14]. Although the content of each specific program may be different, their general aims are for professional growth, personal growth and culture-related personal development [15,16].

Although academics have developed these various learning programs to increase students’ exposure to different cultures [11], their effectiveness may differ. Overseas study programs have been found to have superior effects on cultural competence than in-class learning programs or immersion in cultural-related activities organised in domestic settings [17]. The overseas study programs force students to immerse themselves in another cultural context. During the stay overseas, the programs are designed to ensure students interact with people in that country and immerse themselves in the society at a deep level. However, most of the research investigating the impact of overseas study programs has been limited in their design, for example, particularly when they lack comparison with a control group [18,19] or only assess competence retrospectively [20].

### 1.1. Effects of Overseas Study Experiences on Cultural Competence

Overseas study programs allow students to study abroad and gain insights via cultural immersion in the host country. Before the COVID-19 pandemic, university authorities had dedicated funds to increase cultural competence, and many provide financial support to execute overseas study programs [21]. There is no doubt that long-term programs have some advantages as they allow deeper and richer learning. However, due to financial and practical constraint, these programs are not open to all students. On the contrary, short-term overseas study programs have been argued to contribute to the current cultural education in universities, and have become a popular choice to experience cultural immersion [11]. According to Amerson’s research, even one-week overseas study programs can increase nursing students’ cultural competence [17]. Considering the global trend for expansion of short-term cultural learning programs and the potential effects on cultural competence, it is important to critically evaluate the benefits, or otherwise, of short-term overseas study programs.

A number of studies have explored the effects of short-term overseas study experience on cultural competence. Some studies adopted theories of cultural competence, which did not cover all the four aspects considered in the present study, and hence their evaluation was limited to partial aspects of cultural competence [22,23,24]. However, regardless of whether full or partial competence was measured, it is notable that most studies have found that participants’ cultural competence improved after participation in such programs [22,25]. For example, quantitative measurement in a mixed-method study of healthcare students in the USA reported a significant increase in cultural knowledge and attitude, but not in cultural awareness after a 9-day overseas study program in Ecuador [23]. The participants highlighted achieving personal and professional growth, especially improved cultural competence [15,16]. Despite the positive findings, the validity and reliability of these studies should be considered in terms of methodological issues. There is limited longitudinal research examining the effects of short-term overseas study programs, and mainly qualitative evidence for their effectiveness exists in Australia [25,26,27]. More importantly, the quantitative studies have lacked control for other related factors affecting cultural competence [25,26,27]. Such limitations in research design impaired the quality of these studies, and a well-controlled design is needed.

### 1.2. Essential Factors Affecting Cultural Competence

To evaluate the effectiveness of overseas study programs, it is vital to control for the effects of other essential factors associated with cultural competence. Personality is regarded as an important factor affecting cultural competence, as it determines an individual’s behaviour patterns by affecting one’s intrapersonal processes, such as emotional, motivational, and cognitional processes [28]. Such internal processes influence how people act and feel, and hence personality is what makes a person different or similar to others [29]. Van der Zee and van Oudenhoven identified five personality traits related to intercultural success (i.e., cultural empathy, flexibility, social initiative, emotional stability, and open-mindedness) [30]. However, among various personality traits, open-mindedness has been found to be mostly related to cultural competence [31,32]. Open-mindedness is defined as a personality trait of having an open attitude without prejudice toward people based on cultural differences [30].

Age, gender, education level, and employment status have been found to be associated with attitudes towards multiculturalism, immigration and cultural diversity [33]. Intercultural interaction has also been related to cultural competence [34,35]. However, when considering intercultural interaction after controlling for prior cultural education, the effects of these variables became non-significant for Korean nursing students [36]. Moreover, when considering these demographic variables and intercultural interaction after controlling for open-mindedness and prior overseas study experiences, the effects of these variables became non-significant for Australian healthcare students [32]. Therefore, these variables may not be identified as essential factors affecting cultural competence.

Cultural competence could be enhanced by general education and specific training programs [37]. Contrary to personality, which cannot be easily changed, increasing cultural exposure has been emphasised in cultural learning programs [38]. Prior overseas life experience may influence competence and therefore this should be measured as a control variable [39]. However, recent research has suggested that simply travelling overseas does not contribute significantly to cultural competence after controlling for overseas study experience, which means that the essential factor affecting cultural competence is actually the cultural immersion gained during overseas study programs [32]. Even so, given their confounding potential, prior overseas study experiences should be controlled for when investigating the effects of the overseas study programs on cultural competence.

### 1.3. The Present Study

The objective of the present study was to evaluate the specific effects of short-term overseas study programs on cultural competence among Australian healthcare students. The present study adopted a 2 × 2 (time × group) mixed factorial quasi-experimental design, which has been used in previous literature [22], to conduct an evaluation in a longitudinal manner along with a control for extraneous variables. Cultural competence was measured in a pre- and post-test manner. A control group of students who stayed in Australia to continue their university education as usual, was surveyed as well. Prior overseas study experiences and open-mindedness were measured in the study as control variables. It was hypothesised that after controlling for prior overseas study experiences and the open-mindedness trait, the overseas study groups would have significantly more increases in their cultural competence than the control group.

## 2. Materials and Methods

### 2.1. Participants

Participants were 78 domestic healthcare students from the Queensland University Technology in Australia. They were not first-generation immigrates or international students. The majority of the participants were second- and third-year undergraduate students (see Table 1). The number of students in Group 1 (i.e., overseas study group) was 32, of whom 28 were female, with a mean age of 23.81 (range: 18–49; SD = 5.91). These students were recruited from 11 short-term overseas study programs organised by the Faculty of Health. The programs were open to students from different disciplines including nursing, social work, public health, and nutrition, and hence the participants were from a mixture of disciplines related to healthcare.

Participants in Group 2 (*n* = 46; 42 female) were the fellow students of Group 1, who were enrolled in the same courses, and they were also from various disciplines related to healthcare, but did not go overseas (i.e., control group). These students stayed in Australia to continue their university education. Their ages ranged from 18 to 40 (M = 21.87, SD = 5.08). In the design, Group 2 was set as a control group and the individuals did not attend any specific international cultural learning programs. However, more than half of them reported undertaking in-class cultural learning as part of their domestic health care courses. Since participants were from a mixture of disciplines related to healthcare, they were enrolled in different subjects. For example, nursing students were completing a course about cultural safety; nutrition students were learning about food and culture; public health students were learning about health, culture and society.

### 2.2. Overseas Study Programs

Each student in Group 1 had participated in one of 11 short-term overseas study programs organised by the Faculty of Health in the Australian university. The programs were designed for undergraduate students from disciplines related to healthcare, who were sent to East Asian countries for a short duration (two to four weeks). All programs were located in mainland China, Vietnam, Singapore, or Taiwan. The student experiences were hosted in local universities, hospitals, or other health organisations. These programs were completed before the COVID-19 pandemic.

### 2.3. Measure Instrument

*Instrument to measure demographic information.* A demographic form was developed and utilised in the pre-test. It asked questions about age, gender, perceived sufficiency of family income, religion, ethnicity, and whether they spoke a language other than English. Prior overseas study experiences were measured in the form as well.

*Instrument to measure cultural competence.* The Cultural Intelligence Scale (CQS) by Ang and colleagues was used to measure cultural competence in the pre-test, and again in the post-test [40]. Higher scores indicate a higher level of cultural competence. The CQS was developed based on the theory of cultural intelligence [2]. The CQS has four subscales to measure the metacognitive, cognitive, motivational and behavioural aspects of cultural competence. There are 4 items for cultural awareness (e.g., “I adjust my cultural knowledge as I interact with people from a culture that is unfamiliar to me”); 6 items for cultural knowledge (e.g., “I know the rules for expressing non-verbal behaviours in other cultures”); 5 items for cultural attitude (e.g., “I am confident that I can get accustomed to the shopping conditions in a different culture”), and 5 items for cultural skills (e.g., “I alter my facial expressions when a cross-cultural interaction requires it”). Respondents used a 7-point rating scale, from 1 (strongly disagree) to 7 (strongly agree) to respond to each item. The scale shows good validity and reliability (Cronbach’s alpha 0.70–0.88) with both USA and Singaporean samples [40]. The CQS was originally developed in both English and Chinese, and the English version was used in the present study.

*Instrument to measure open-mindedness.* The open-minded subscale in the Multicultural Personality Questionnaire-short form (MPQ-SF) by van der Zee and van Oudenhoven was used in the pre-test [41,42]. The subscale has eight items and an example item is “I am a person who tries out various approaches”. The scale shows sufficient psychometric properties and this subscale has good internal consistency (α = 0.72) [42]. Respondents use a 5-point rating scale (1 = totally not applicable, 5 = completely applicable). The English version of MPQ-SF from literature was used and no translation was conducted [42].

### 2.4. Research Procedure: Ethical Approval and Participants’ Recruitment

Ethical approval was obtained from the University Human Research Ethics Committee (Ethics Approval Number: 1600000806). All study participants were informed about the purpose, risks and benefits of the study. The questionnaires were administered by the lead author.

To recruit Group 1, the lead researcher went to the pre-departure briefing seminars. Students completed the pre-test (about 15 min) during the seminar time, two to six weeks before their departure. After they completed the overseas trips, they were asked to carry out the post-test (about 10 min). The post-test was conducted two to eight weeks after their return from overseas. On average, the time interval between pre- and post-test was 10 weeks.

To recruit Group 2, the lead researcher went to lectures and tutorials which students in Group 1 were enrolled in, to access the population pool for the control group. The researcher went to these classes twice during and at the end of the semester to conduct the pre- and post-tests. The time interval between pre- and post-test was consistent with that of Group 1. Only those who were domestic undergraduate students, and had not enrolled in any short-term overseas study programs in the coming three months, were invited to take part in the research.

### 2.5. Statistical Analysis

SPSS 22.0 was used in the statistical analysis [43]. Group differences at baseline were examined by conducting t-tests for the continuous data and chi-square tests for the categorical data. Additionally, ANCOVAs were conducted to explore the different changes of cultural competence between groups over time, with prior overseas study experiences and open-mindedness as the covariates.

## 3. Results

### 3.1. Demographic Data

Demographic data are presented in Table 1. Potential group differences on the pre-trip baseline were checked via t-tests and chi-square tests. There were no significant differences for age *t*(76) = 1.55, *p* = 0.125, gender *χ*^2^ (1, *n* = 78) = 0.29, *p* = 0.586, university year level *χ*^2^ (3, *n* = 78) = 4.22, *p* = 0.239, religious beliefs *χ*^2^ (1, *n* = 76) = 0.03, *p* = 0.866, ethnicity *χ*^2^ (1, *n* = 78) = 0.87, *p* = 0.352, second language *χ*^2^ (1, *n* = 78) = 0.74, *p* = 0.390, family language *χ*^2^ (1, *n* = 78) = 0.06, *p* = 0.804, or perceived income sufficiency between groups *t*(50.53) = 1.26, *p* = 0.213.

### 3.2. Data of Cultural Competence

The means and standard deviations on cultural competence are presented in Table 2. The scale scores indicate the two groups’ level of cultural competence in the pre- and post-test. Although the groups were equivalent on the demographics, they did not show the same level of cultural competence at baseline. Specifically, the two groups shared the same pre-test scores on awareness *t*(76) = 1.50, *p* = 0.138, *d* = 0.35, knowledge *t*(76) = 1.10, *p* = 0.276, *d* = 0.26 and skills t(76) = 1.68, *p* = 0.098, *d* = 0.39, but significantly different scores on attitude *t*(76) = 2.73, *p* = 0.008, *d* = 0.65, which contributed to the significantly different overall scores of cultural competence, t(76) = 2.27, *p* = 0.026, *d* = 0.53.

### 3.3. ANCOVA Analysis

**Figure 1:**
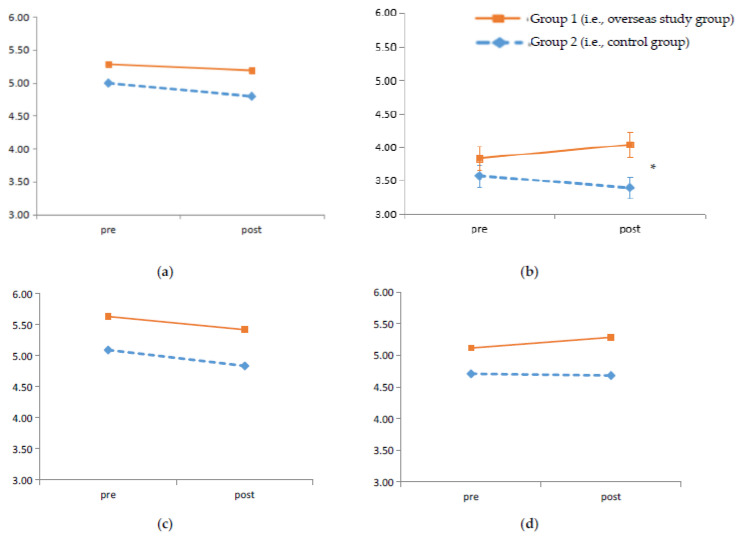
Levels of cultural competence in pre- and post-test. The time interval between the pre- and post-test was about 10 weeks. Error bars represent ± 1 SE. * indicates a significant interaction effect (*p* < 0.05). (**a**) Metacognitive aspects: cultural awareness; (**b**) Cognitive aspects: cultural knowledge; (**c**) Motivational aspects: cultural attitude; (**d**) Behavioural aspects: cultural skills.

To compare the changed tendency of cultural competence between groups over time, 2 × 2 (time × group) mixed factorial ANCOVAs were conducted, with open-mindedness and prior overseas experiences as the covariates (see Figure 1). All the assumptions of ANCOVAs were met.

The results of ANCOVAs are presented in Table 3. The main effect of time was not significant. However, a decreased tendency of cultural competence over time was noted, especially for the control group. The data revealed that students tended to report lower cultural competence in the post-test, compared to the pre-test.

As for the time × group interaction, the effect was significant on cultural knowledge (*p* < 0.05) but not on the cultural awareness, attitudes or skills (*p* > 0.05) in Group 1, and a there was a trend for an increase in cultural skills. Considering the limited number of participants, effect size was examined as supportive evidence. The interaction effect on cultural knowledge had a medium effect size (*η*^2^ = 0.06). The results suggest that, compared to the students in the control group, students had a significant increase in their cultural knowledge after undertaking the overseas study programs. However, there was no significant difference on the other aspects of cultural competence.

## 4. Discussion

### 4.1. Effect of Short-Term Overseas Study Programs on Cultural Competence

The overseas study group had significantly increased scores in cultural knowledge following their study programs, when compared to the control group, which partially supported the main hypothesis. In other words, if a student chose to take part in a short-term overseas study program, they would obtain more cultural knowledge than their fellow students who did not. Several quantitative studies have indicated positive but limited effects of short-term overseas study programs on cultural competence [44,45,46,47]. Some studies did not find a significant increase in cultural awareness [46], attitude [48], or skills [47] following short-term overseas study programs. Improvement in the cognitive aspects of cultural competence after short-term overseas study programs, found in the present study, is consistent with previous research [19,48]. For example, a survey suggested a large enhancement of cultural knowledge for nursing students, though no inferential statistics were conducted [17].

Compared with the control group, the overseas group demonstrated a higher level of cultural competence, especially cultural attitude and skills in the pre-test. According to these differences, it could be inferred that the students who tend to study overseas have a desire to know a new culture and seek cultural exposure compared to those who choose not to participate in such programs. This finding is aligned with the previous literature. Driven by desire, these students who choose to travel might frequently obtain cultural information from multiple sources and take more in-class cultural learning opportunities. Conversely, students who are not likely to study overseas may lack the desire or be obstructed in forming a desire to learn more about other cultures. The prospect of spending several weeks far away from home independently could be perceived as overwhelming [49]. It is important to note that the present study was conducted before the COVID-19 pandemic, and thus the specific difficulties encountered by overseas programs related to the pandemic, such as depression, anxiety, and sleep disorders [50], are beyond the scope of this paper.

### 4.2. Decreased Scores of Cultural Competence

It was noted that there was a tendency of decreased cultural competence over time, that is, the overseas study group’s scores of cultural awareness and attitude decreased. Due to this unexpected tendency, a question was raised as to whether students decreased their cultural competence after learning. It may be that students had a tendency to decrease their perceptions of their own cultural competence when realising how much more they had to learn.

Consistently, in a study with 121 baccalaureate nursing students, participants rated themselves higher in the pre-test than in the post-test on several items. It was suggested that students had their own worldview prior to the experience and they probably realised that their previous worldview or assumptions were limited after the overseas experiences [48]. So, the overseas study experiences helped students with expanding and/or modifying their previous worldview, assumptions and understanding. The decreased score was likely to be a reflection of the clearer understanding of one’s cultural competence, or lack thereof. Based on the theory proposed by Campinha-Bacote, being culturally competent is a long journey, and realising oneself is not really competent enough is also progress on the journey [5]. Furthermore, a recent longitudinal study on Australian nursing students also suggested a decreasing pattern on the post-test immediately after overseas study, whereas one-year-later they showed increased scores on cultural competence [19]. This longitudinal data-based evidence also supported that the decreasing scores could indicate another form of developed cultural competence.

Additionally, the students in the control group rated themselves lower on cultural competence over time as well, although this did not reach statistical significance. This is consistent with a small number of studies, which found a non-significant effect on cultural competence in students who participated in in-class cultural learning programs [51,52]. Assuming the change would be significant with a larger sample size, a potential explanation for this is that these participants were all from the Faculty of Health, and most of them were undertaking cultural learning courses because cultural learning has been integrated into the education curriculum of healthcare students. Along with the cultural learning in class, these students can still expand and/or modify their previous worldview, assumptions and understanding while at university. The cultural learning at university developed their understanding of cultural issues, and their improved cultural competence may have led them to believe they were far away from being truly culturally competent. Therefore, the scores were decreased as a result of a clearer perception the students had of themselves.

### 4.3. Theoretical and Practical Implications

As shown in the literature, short-term overseas study programs can improve the cultural competence of students in the USA [22,53] and nursing students in Australia [25,26,27]. The present study added to these findings using a well-controlled research design. Additionally, the present study has highlighted that the immediate improvement on cultural competence caused by short-term overseas study programs is limited to cognitive aspects. Theoretically, cognition might be in the first layer of cultural competence, and hence could be most sensitive to the intervention.

Short-term overseas study programs are good options for educators looking to develop healthcare students’ cultural knowledge. Nevertheless, having a better understanding of themselves and complex cross-cultural situations is also an authentic contribution of the programs to cultural competence.

### 4.4. Limitations and Future Directions

The research has several limitations. The sample, though there were more than 30 participants in each group, was not large and was dominated by females. It is important to note that there are more female students enrolled in health sciences. The study was also impacted by selection bias as the participants in the overseas programs displayed higher cultural attitude initially than the participants in the control group. Selection bias is a common limitation that can hardly be avoided in this research field [20]. Further, the study focused on the students’ perceptions soon after completion of their programs. It is unknown whether the programs have longer term impacts, as has been shown elsewhere [54,55].

Future studies should recruit larger gender balanced samples form a variety of university courses and disciplines. Larger samples from multiple disciplines could help in overcoming the selection bias. Similarly, continued follow-up studies are warranted to shed light on how long students maintained the observed changes in cultural competence.

## 5. Conclusions

The research revealed that short-term overseas study programs could enhance healthcare students’ cultural competence, specifically cultural knowledge. Further studies are needed in the future to examine the lasting effect of these short-term overseas study programs.

## Figures and Tables

**Table 1:** Demographic characteristics of the sample.

Variable	Group 1 ^1^		Group 2 ^2^	
	*n*	(%)	*n*	(%)
Age				
M ± SD	23.81 ± 1.04		21.87 ± 0.75	
Gender				
Male	4	(12.5)	4	(8.7)
Female	28	(87.5)	42	(91.3)
University year level				
1st	0	(0.0)	3	(6.5)
2nd	20	(62.5)	21	(45.7)
3rd	9	(28.1)	19	(41.3)
4th	3	(9.4)	3	(7.5)
Religion				
None	18	(56.3)	27	(58.7)
Yes	13	(40.6)	18	(39.1)
Ethnicity				
Ethnic minority	13	(40.6)	14	(30.4)
Ethnic majority (Caucasian)	19	(59.4)	32	(69.6)
Speak another language except for English				
No	20	(62.5)	33	(71.7)
Yes	12	(37.5)	13	(28.3)
Language spoken in family				
English	25	(78.1)	37	(80.4)
Others	7	(21.9)	9	(19.6)
Income sufficiency				
M ± SD	3.63 ± 0.18		3.89 ± 0.10	

^1^ Group 1 = overseas study group; ^2^ Group 2 = control group.

**Table 2:** Descriptive statistics (mean ± SD) of cultural competence.

	Pre-Test		Post-Test	
	Group 1 ^1^ (*n* = 32)	Group 2 ^2^ (*n* = 46)	Group 1 (*n* = 32)	Group 2 (*n* = 46)
Metacognitive-Cultural awareness	5.28 ± 0.72	4.99 ± 0.90	5.19 ± 1.14	4.79 ± 0.87
Cognitive-Cultural knowledge	3.83 ± 0.97	3.57 ± 1.09	4.04 ± 1.05	3.39 ± 1.12
Motivational-Cultural attitude	5.63 ± 0.66	5.09 ± 0.97	5.42 ± 1.10	4.83 ± 1.00
Behavioural-Cultural skills	5.12 ± 1.02	4.71 ± 1.09	5.29 ± 1.20	4.68 ± 0.90
Total cultural competence	4.97 ± 0.64	4.59 ± 0.77	4.98 ± 1.04	4.43 ± 0.77

^1^ Group 1 = overseas study group; ^2^ Group 2 = control group.

**Table 3:** Results of ANCOVAs.

	Time		Group		Time × Group		Personality		Prior Overseas Study	
	*F*	*p*	*F*	*p*	*F*	*p*	*F*	*p*	*F*	*p*
Metacognitive-Cultural awareness	0.15	0.691	1.13	0.290	0.32	0.572	30.15	<0.001	1.73	0.192
Cognitive-Cultural knowledge	0.002	0.961	1.94	0.168	4.54	0.037	16.51	<0.001	0.42	0.521
Motivational-Cultural attitude	0.14	0.705	4.71	0.033	0.07	0.799	51.24	<0.001	1.03	0.314
Behavioural-Cultural skills	0.54	0.464	4.54	0.036	0.27	0.604	3.22	0.077	0.13	0.715

## Data Availability

Without using any open data, the present study is part of the PhD research project completed by the leading author at QUT. Raw data have been archived in the QUT storage.
